# Current-future burden concerning musculoskeletal diseases and high body mass index

**DOI:** 10.1097/MD.0000000000050724

**Published:** 2026-09-18

**Authors:** Jiantong Wei, Qingqing Qin, Hao Chen, Wenqiang Liang, Yaohua Wang

**Affiliations:** a Department of Orthopedics, Zhangye People’s Hospital Affiliated to Hexi University, Zhangye, China; b The First Clinical Medical College of Lanzhou University, Lanzhou, China.

**Keywords:** body mass index, disability-adjusted life years, disorder burden, inequality, musculoskeletal disorders, trends

## Abstract

This study aims to systematically assess the global burden of musculoskeletal (MSK) diseases caused by high body mass index (BMI) and to analyze its temporal trends and cross-national inequalities. Data were extracted from the Global Burden of Disease 2021. We quantified the temporal trends by calculating the estimated annual percentage change of the age-standardized DALYs rate (ASDR). Joinpoint regression analysis was used to identify the significant turning points in the ASDR. The cross-national inequality situation was evaluated using the sociodemographic index (SDI). The Bayesian Age-Period-Cohort model was constructed to predict the disease burden up to 2050. Over the past 30 years, the ASDR for MSK diseases attributable to high BMI increased substantially from 110.57 per 100,000 in 1990 to 155.33 per 100,000 in 2021. In 2021, the ASDR of MSK diseases attributable to high BMI was highest in areas with high SDI and lowest in areas with low SDI, and there was a clear positive association between the ASDR of MSK diseases attributable to high BMI and SDI levels. By sex and age, the burden of MSK disease attributable to high BMI is much higher in women than in men, and disability-adjusted life years cases of MSK disease attributable to high BMI peak in the 55 to 59 age group, globally. Cross-country inequality analysis showed that there were clear absolute and relative SDI-related inequalities in disability-adjusted life years of MSK disease attributable to high BMI in 204 countries and regions, with countries and regions with higher SDI bearing a disproportionate burden. In addition, the global burden of MSK disease attributable to high BMI is likely to increase further between 2022 and 2050. Since 1990, the global burden of MSK diseases attributable to high BMI has been clearly rising. The burden of MSK disease attributable to high BMI varies significantly across countries, regions, genders, and ages. Public health strategies that address obesity risk factors are critical to reducing the growing burden of MSK disease.

## 1. Introduction

Musculoskeletal (MSK) diseases are a combination of muscle, bone, joint, or adjacent connective tissue damage, which may lead to short-term or lifelong limitations in human function and participation.^[1,2]^ The incidence and prevalence of MSK disorders have been increasing globally for many years, placing a heavy economic and physical burden on patients.^[3]^ The development of MSK disorders is exacerbated by the fact that people are often engaged in repetitive movements for long periods, resulting in poor working posture, as well as insufficient rest and recovery time.^[4]^ Some studies have shown that aging is strongly associated with the development of MSK disorders, with the onset of MSK disorders becoming more likely with increasing age.^[5,6]^ In addition, a high body mass index (BMI) is also strongly associated with the development of MSK diseases, and the rapid increase in the number of people with a high BMI is an important reason for the dramatic increase in the burden of MSK diseases in recent years.^[7]^

Overweight and obesity have become a serious public problem, with a substantial increase in high BMI-related deaths and disability-adjusted life years (DALYs) globally from 1990 to 2021.^[8]^ Epidemiologic studies have found that a high BMI is a major driver in the development of MSK disorders.^[9]^ One study found a strong relationship between the intensity of pain from MSK disorders and a high BMI.^[10]^ Other studies found that a high BMI was a significant risk factor for years of life lived with disability resulting from MSK disorders.^[11,12]^ Previous studies showed that factors such as high BMI, smoking, and occupational ergonomic factors are risk factors for MSK disorders, with a high BMI ranking first among all risk factors.^[11]^

Based on the latest data from the Global Burden of Disease (GBD) 2021, we conducted a comprehensive analysis of the DALYs of MSK disorders attributable to a high BMI at global, regional, and country levels from 1990 to 2021, comparing the distribution and changes in the burden of MSK diseases across different regions, countries, genders, and age groups, with the aim of understanding the global prevalence characteristics of MSK diseases attributable to a high BMI and providing some reference value for policymaking on the prevention and treatment of MSK diseases.

## 2. Materials and methods

### 2.1. Data sources

Data for this study were obtained from the GBD 2021 database, which provides information on the global and regional burdens of 371 diseases, injuries, and 88 risk factors for 204 countries and territories from 1990 to 2021. We extracted the corresponding data for MSK disorders attributable to a high BMI. In GBD 2021, a high BMI is defined as obese individuals with a BMI of more than 25 kg/m^2^. The sociodemographic index (SDI) is used to quantify the level of development of a country or region using data on fertility, education level, and per capita income. The SDI takes values ranging from 0 to 1, with higher values indicating better socioeconomic development. The relationship between the SDI and the disease burden in 2021 was analyzed by calculating the disease rate of the different SDI regions. SDI categorization (low, low-middle, middle, high-middle, and high) was used to compare the burden of disease at different levels of socioeconomic development.

The study adhered to the Guidelines for Accurate and Transparent Reporting of Health Estimates. The study did not involve any personal or sensitive information. Therefore, no ethical approval was required to perform this study.

### 2.2. Data cleaning and statistical analysis

Temporal patterns in age-standardized DALYs rate (ASDR) for MSK disorders attributable to a high BMI were quantified using the estimated annual percentage change (EAPC). In epidemiological studies, EAPC is used to assess the changes in disease rates over time.^[13]^ When the lower limit of the 95% confidence interval (CI) exceeds 0, it indicates an upward trend, and the opposite indicates a downward trend. If the 95% CI contains 0, it indicates no statistically significant change in the trend pattern.

Draw global maps depicting the burden of MSK diseases caused by high BMI to clearly present the distribution and regional differences of the MSK disease burden caused by high BMI at the regional and national levels (the ArcGIS World Vector Atlas provides global vector map data, which is used for precise matching with the statistics of GBD for spatial analysis). To compare the burden of MSK diseases attributable to high BMI at different levels of socioeconomic development, the SDI classification (low, low-middle, middle, high-middle, and high) was used to compare the burden of disease at different levels of socioeconomic development, and correlation analyses were conducted to clarify the relationship between SDI levels and the burden of MSK diseases attributable to high BMI.^[14]^ To explore the distribution of MSK disorders attributable to high BMI among different genders and age groups, we stratified males and females into 16 age groups, including 20 to 24, 55 to 59, 60 to 64, 65 to 69, and 70 to 74 years.

To quantify differences in the distribution of MSK disorders attributable to high BMI across countries and regions and to provide a comprehensive analysis and assessment of health inequalities, we compared data from 204 countries and regions from 1990 to 2021 through cross-national inequality analysis.^[15]^ To predict the future burden of MSK disorders attributable to high BMI, we applied Bayesian Age-Period-Cohort modeling (BAPC).^[15]^ BAPC has been shown to have higher coverage and accuracy than other prediction methods.

All analyses and visualizations were performed using R software (version 4.4.2; R Foundation for Statistical Computing) and Joinpoint software (version 5.3.0; National Cancer Institute). All analyses were performed using appropriate statistical models, and *P* < .05 was considered statistically significant.

## 3. Results

### 3.1. Burden of MSK disorders attributable to high BMI

**Figure 1.**
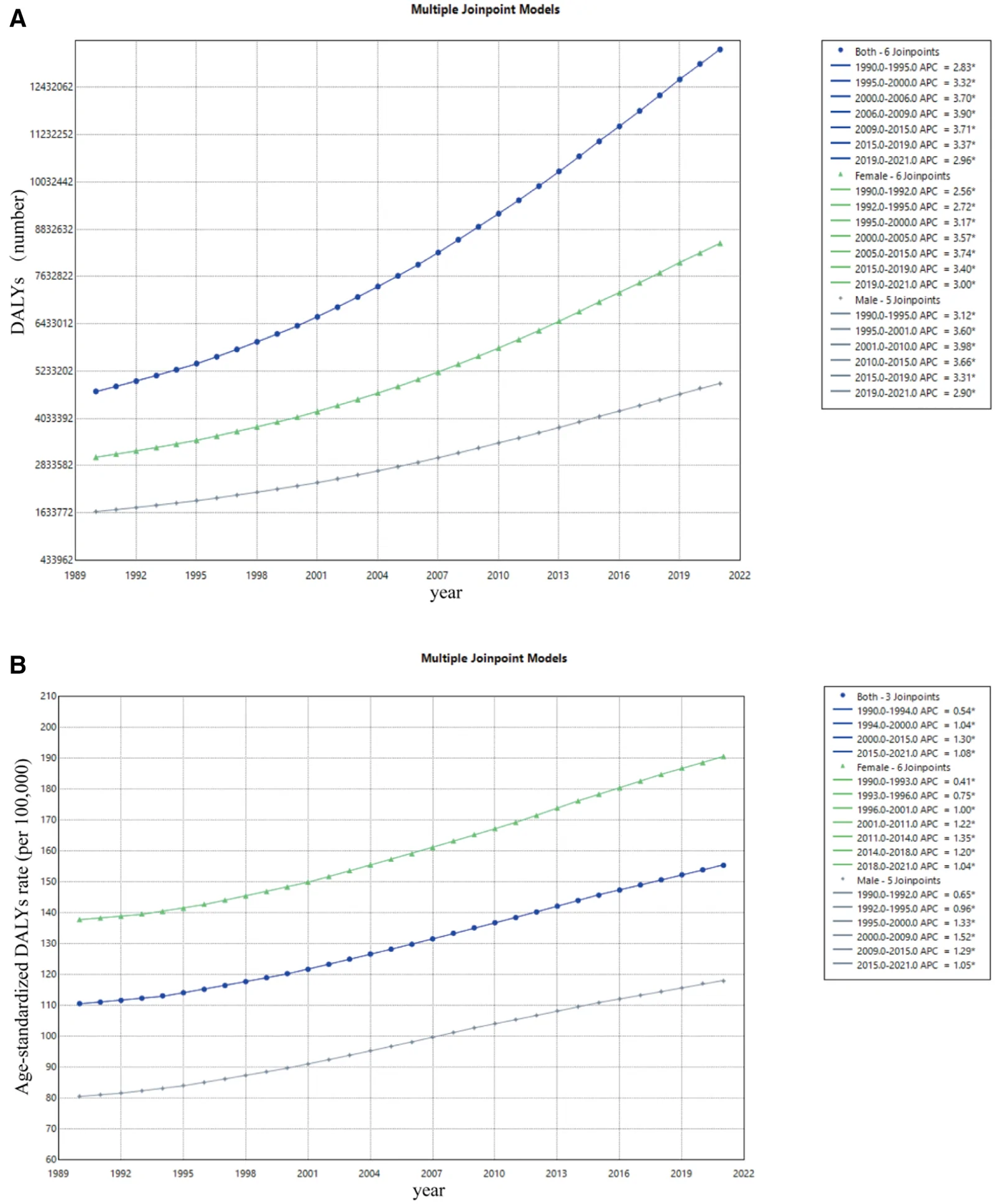
Global temporal trends of MSK disease burden attributable to high BMI from 1990 to 2021. (A) Number of DALYs. (B) Age-standardized DALYs rates. BMI = body mass index, DALYs = disability-adjusted life years, MSK = musculoskeletal.

Globally, the number of DALYs for MSK disorders attributable to high BMI increased substantially from 4,717,812 cases in 1990 to 13,400,010 cases in 2021. The ASDR increased from 110.57 per 100,000 in 1999 to 155.33 per 100,000 in 2021. The ASDR of MSK disorders attributed to high BMI showed a significant upward trend from 1990 to 2021, with an EAPC of 1.18 (95% CI = 1.15–1.21; Table 1 and Fig. 1). In addition, we found that the ASDR of MSK disorders attributable to high BMI increased in all 5 SDI regions, with the largest increase in the low-middle SDI region, with an EAPC of 2.30 (95% CI = 2.25–2.35). In 2021, the ASDR of MSK disorders attributable to high BMI was highest in the high SDI region and lowest in the low SDI regions (Table 1).

**Table 1:** DALYs of MSK diseases attributable to high BMI at global and regional levels from 1990 to 2021.

Location	1990	ASDR (95% UI)	2021	ASDR (95% UI)	EAPC (95% CI)
	Number (95% UI)		Number (95% UI)		
Global	4,717,812 (371,436–10,245,415)	110.57 (8.37–240.46)	13,400,010 (1,048,084–28,718,885)	155.33 (12.24–332.41)	1.18 (1.15–1.21)
High SDI	1,868,991 (147,787–4,054,642)	179.17 (14.51–389.65)	3,989,787 (344,444–8,265,577)	242.06 (22.50–499.86)	1.02 (1.00–1.03)
High-middle SDI	1,397,649 (112,683–3,016,884)	135.51 (10.73–291.75)	3,337,956 (252,172–7,151,544)	179.15 (14.10–383.29)	0.97 (0.92–1.01)
Middle SDI	901,032 (67,358–2,001,881)	73.65 (5.02–167.64)	3,726,107 (263,041–8,177,518)	132.81 (9.38–290.77)	2.06 (2.01–2.10)
Low-middle SDI	414,740 (32,463–903,704)	56.38 (4.07–124.01)	1,808,079 (143,745–3,850,182)	109.62 (8.31–234.28)	2.30 (2.25–2.35)
Low SDI	127,944 (10,307–272,138)	46.37 (3.47–101.53)	523,687 (43,403–1,100,759)	79.86 (6.11–170.87)	1.82 (1.76–1.87)
Andean Latin America	25,951 (1431–57,989)	108.59 (5.28–246.73)	107,006 (6212–234,062)	169.87 (9.49–370.16)	1.50 (1.47–1.53)
Australasia	51,253 (4690–111,237)	226.32 (20.85–492.40)	140,553 (13,812–289,743)	327.04 (32.87–675.34)	1.23 (1.14–1.32)
Caribbean	30,206 (1706–67,613)	107.65 (5.66–242.11)	83,972 (4508–182,289)	158.81 (8.67–344.03)	1.32 (1.28–1.37)
Central Asia	79,320 (6807–169,037)	158.65 (13.53–337.86)	182,076 (15,723–376,692)	199.69 (17.28–416.30)	0.76 (0.75–0.77)
Central Europe	317,766 (25,631–675,301)	216.67 (17.49–458.89)	500,523 (40,181–1,049,624)	273.88 (22.41–566.98)	0.75 (0.72–0.78)
Central Latin America	151,326 (9773–333,278)	146.79 (8.24–326.72)	566,595 (34,867–1,213,964)	215.90 (13.06–463.22)	1.25 (1.22–1.28)
Central Sub-Saharan Africa	13,928 (1109–29,670)	50.59 (3.67–110.11)	76,473 (6503–161,130)	105.35 (8.17–222.97)	2.36 (2.31–2.41)
East Asia	548,311 (40,877–1,283,481)	55.65 (3.82–131.16)	2,482,737 (163,756–5,769,034)	114.08 (7.82–265.02)	2.68 (2.55–2.82)
Eastern Europe	559,550 (45,860–1,189,205)	204.41 (16.79–430.49)	852,475 (70,983–1,767,192)	269.04 (22.94–556.38)	0.98 (0.95–1.02)
Eastern Sub-Saharan Africa	46,704 (3900–99,569)	49.99 (3.83–109.27)	202,143 (17,315–422,549)	88.84 (6.92–189.43)	1.89 (1.86–1.91)
High-income Asia Pacific	188,233 (12,767–435,315)	91.93 (6.26–211.59)	414,639 (24,711–955,749)	122.98 (8.67–275.90)	0.95 (0.91–0.99)
High-income North America	833,762 (71,180–1,812,467)	258.38 (22.62–562.32)	1,772,685 (184,206–3,511,621)	339.29 (37.14–666.33)	0.96 (0.92–0.99)
North Africa and Middle East	311,604 (25,989–676,071)	149.65 (11.67–324.77)	1,373,607 (121,109–2,829,173)	246.41 (20.83–505.85)	1.63 (1.62–1.65)
Oceania	4276 (371–9287)	109.84 (8.75–239.28)	13,892 (1206–29,803)	140.41 (11.30–301.47)	0.78 (0.72–0.84)
South Asia	244,216 (18,920–538,275)	34.25 (2.45–77.08)	1,279,106 (98,241–2,732,607)	76.58 (5.67–164.03)	2.86 (2.76–2.96)
Southeast Asia	119,146 (11,141–258,699)	37.96 (3.30–84.26)	577,873 (50,927–1,247,588)	78.49 (6.77–169.67)	2.50 (2.43–2.57)
Southern Latin America	92,773 (7555–200,871)	197.45 (16.07–427.15)	229,284 (19,358–478,459)	282.76 (24.24–590.85)	1.18 (1.13–1.23)
Southern Sub-Saharan Africa	43,379 (3529–92,107)	139.75 (10.70–297.97)	130,039 (10,929–267,895)	196.37 (15.66–404.92)	1.12 (1.10–1.14)
Tropical Latin America	155,137 (10,715–349,688)	142.29 (8.90–321.81)	579,685 (38,047–1,256,882)	222.22 (14.56–480.19)	1.45 (1.42–1.48)
Western Europe	823,398 (58,043–1,806,773)	159.53 (11.89–347.82)	1,517,202 (104,932–3,277,294)	212.43 (16.21–457.70)	0.94 (0.91–0.98)
Western Sub-Saharan Africa	77,571 (6139–166,357)	74.46 (5.47–162.79)	317,445 (25,184–676,434)	123.70 (8.80–268.67)	1.61 (1.57–1.65)

ASDR = age-standardized DALYs rate, BMI = body mass index, DALYs = disability-adjusted life years, EAPC = estimated annual percentage change, MSK = musculoskeletal, SDI = sociodemographic index, UI = uncertainty interval.

Regionally, all 21 GBD regions showed increases in the ASDR of MSK disorders attributed to high BMI from 1990 to 2021, with the largest increase in South Asia (EAPC = 2.86; 95% CI = 2.76–2.96) and the lowest growth seen in Central Europe (EAPC = 0.75; 95% CI = 0.72–0.78; Table 1). In 2021, the ASDR of MSK disorders attributed to high BMI was highest in high-income North America (339.29 per 100,000), followed by Australasia (327.04 per 100,000). In contrast, the ASDR of MSK disorders attributed to high BMI was lowest in South Asia (76.58 per 100,000), followed by Southeast Asia (78.49 per 100,000) in 2021 (Table 1).

**Figure 2.**
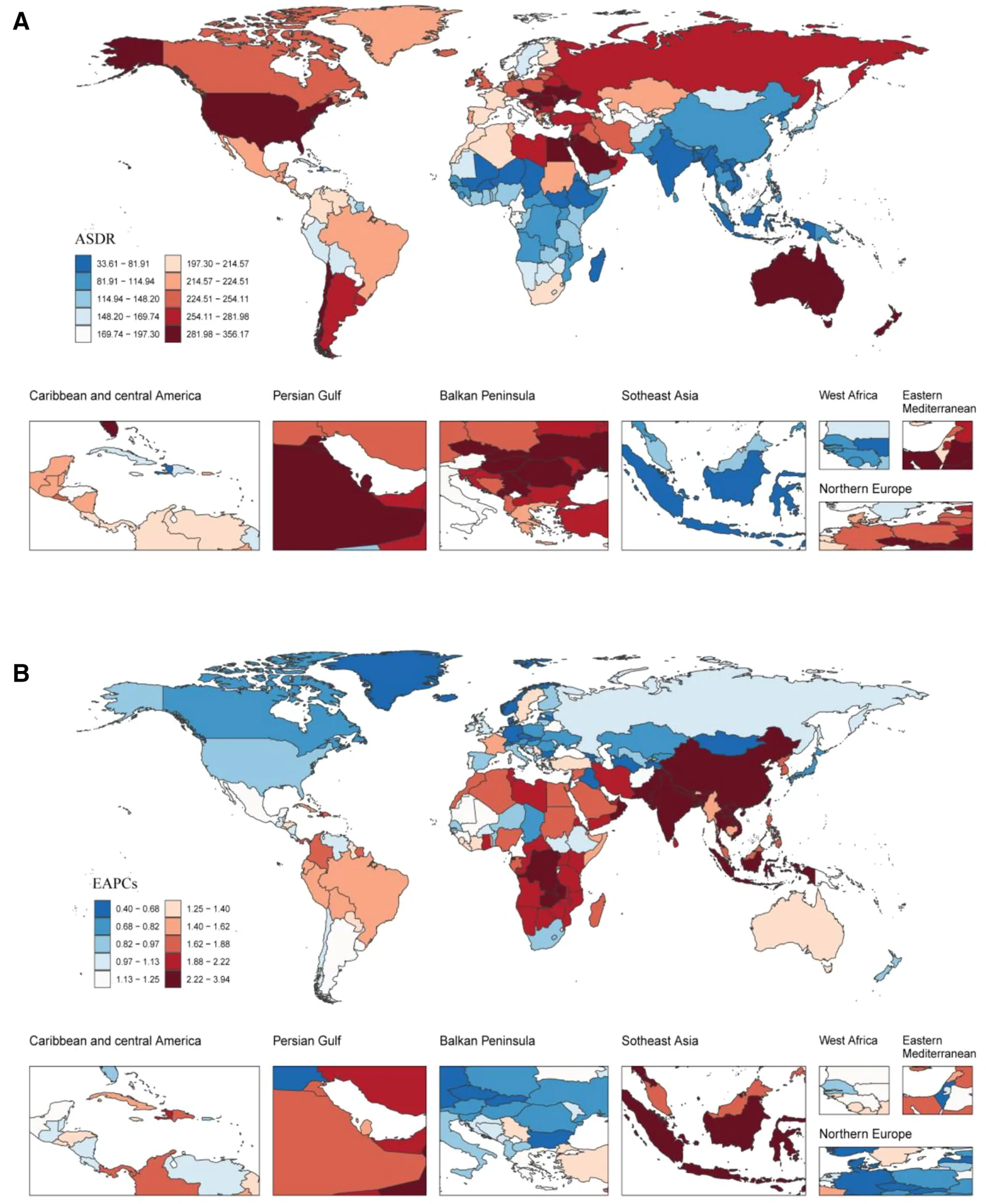
Global burden of MSK diseases attributable to high BMI. (A) ASDR of MSK diseases attributable to high BMI in 2021. (B) EAPCs of MSK disorder DALYs attributable to high BMI from 1990 to 2021. ASDR = age-standardized DALYs rate, BMI = body mass index, DALYs = disability-adjusted life years, EAPC = estimated annual percentage change, MSK = musculoskeletal.

At the country level, the United States of America, Hungary, and Australia were the 3 countries with the highest ASDR of MSK disorders attributed to high BMI in 2021. In contrast, South Sudan, Vietnam, and Timor-Leste were the 3 countries with the lowest ASDR of MSK disorders attributed to high BMI (Fig. 2A). Bangladesh, Vietnam, and Pakistan were the 3 countries with the largest increases in ASDR of MSK disorders attributed to high BMI between 1990 and 2021. In contrast, Czechia, Slovakia, and Georgia were the 3 countries with the lowest increases in ASDR of MSK disorders attributed to high BMI (Fig. 2B).

### 3.2. Association between SDI levels and the burden of MSK disease attributed to high BMI

**Figure 3.**
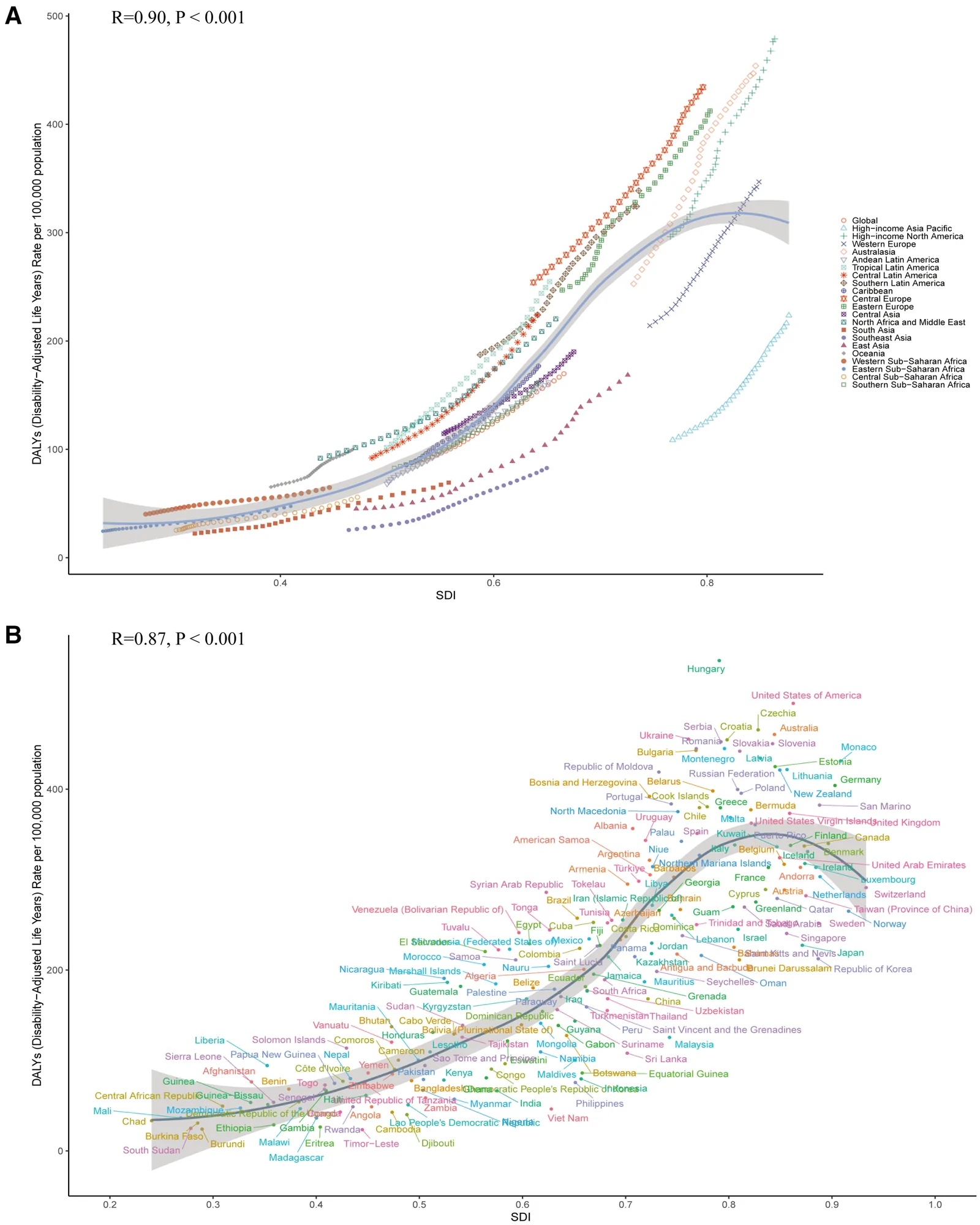
Associations between SDI and DALYs of MSK diseases attributable to high BMI in the 21 GBD regions and 204 countries and territories worldwide. (A) Association between DALYs rates and SDI levels in the 21 GBD regions. (B) Association between DALYs rates and SDI levels in 204 countries and territories. BMI = body mass index, DALYs = disability-adjusted life years, GBD = Global Burden of Diseases, MSK = musculoskeletal, SDI = sociodemographic index.

SDI is a composite indicator used to assess regional and national levels of development and is intricately linked to the health status of local populations. Our findings suggest that the burden of MSK diseases attributable to high BMI is strongly associated with SDI levels. We observed a significant positive association between SDI levels and the ASDR of MSK disorders attributable to high BMI in 21 GBD regions (*R* = 0.90, *P* < .001; Fig. 3A). Similarly, we observed a significant positive correlation between SDI levels and ASDR for MSK disorders attributed to high BMI in 204 countries and territories (*R* = 0.87, *P* < .001; Fig. 3B).

### 3.3. Burden of MSK disease attributed to high BMI by sex and age

**Figure 4.**
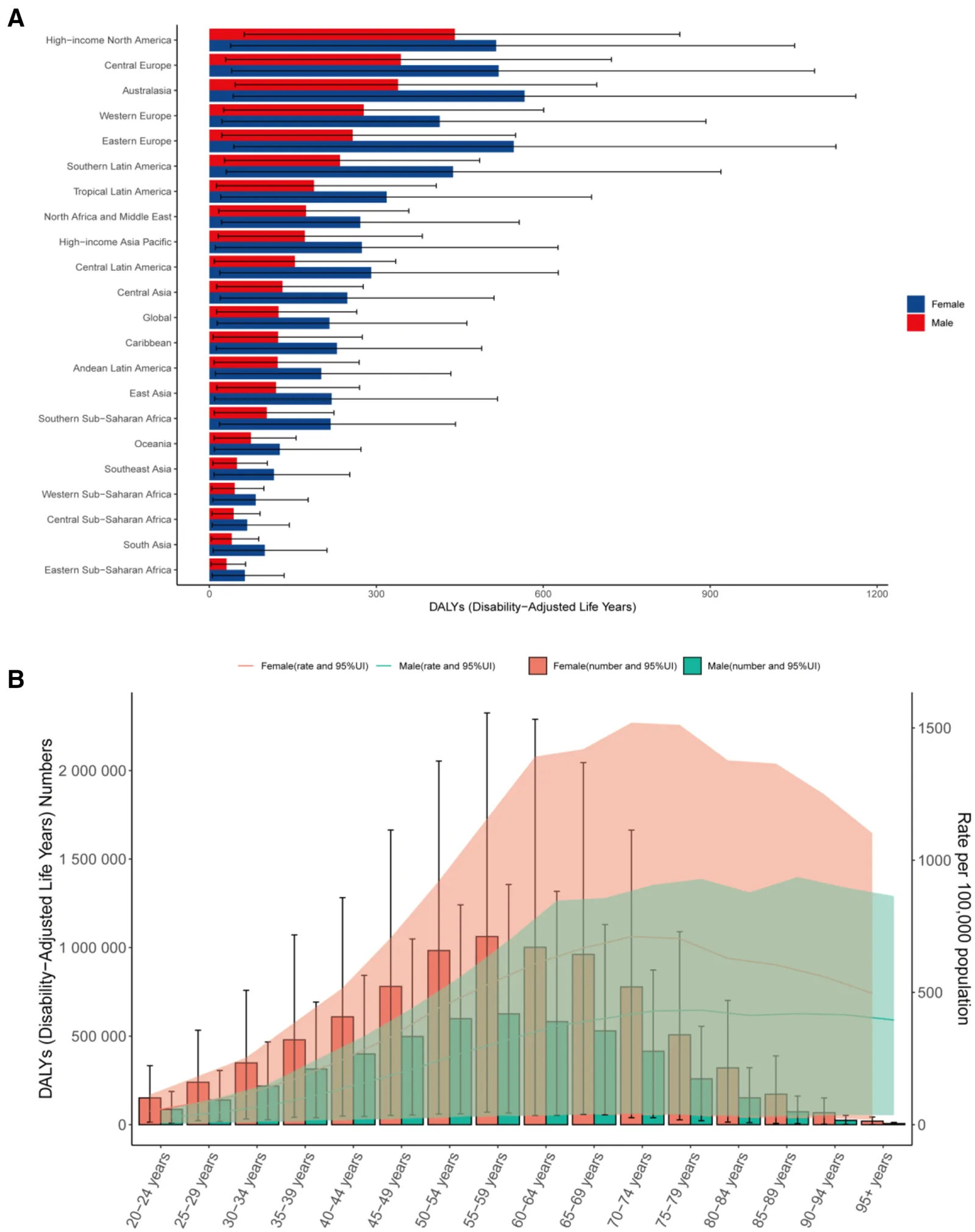
DALYs of MSK diseases attributable to high BMI by sex and age group in 2021. (A) Gender profile of MSK disorders attributed to high BMI in the 21 GBD region. (B) Gender and age profile of MSK diseases attributed to high BMI globally. BMI = body mass index, DALYs = disability-adjusted life years, GBD = Global Burden of Diseases, MSK = musculoskeletal.

From 1990 to 2021, the increase in the ASDR of MSK disorders attributable to high BMI was significantly higher in women than in men, and the DALYs of MSK disorders attributable to high BMI were significantly higher in women than in men (Fig. 1). In 2021, globally and within the 21 GBD regions, the DALYs of MSK disorders attributable to high BMI were significantly higher in women than in men (Fig. 4A). By age group, the DALYs rates of MSK disorders attributable to high BMI first showed an increasing trend with age and then a decreasing trend after reaching a peak. Across all age groups, DALYs of MSK disorders attributable to high BMI were significantly higher in women than in men, and DALYs of MSK disorders attributable to high BMI peaked in the 55 to 59 age group (Fig. 4B).

### 3.4. Cross-national inequality analysis of MSK disease attributable to high BMI

**Figure 5.**
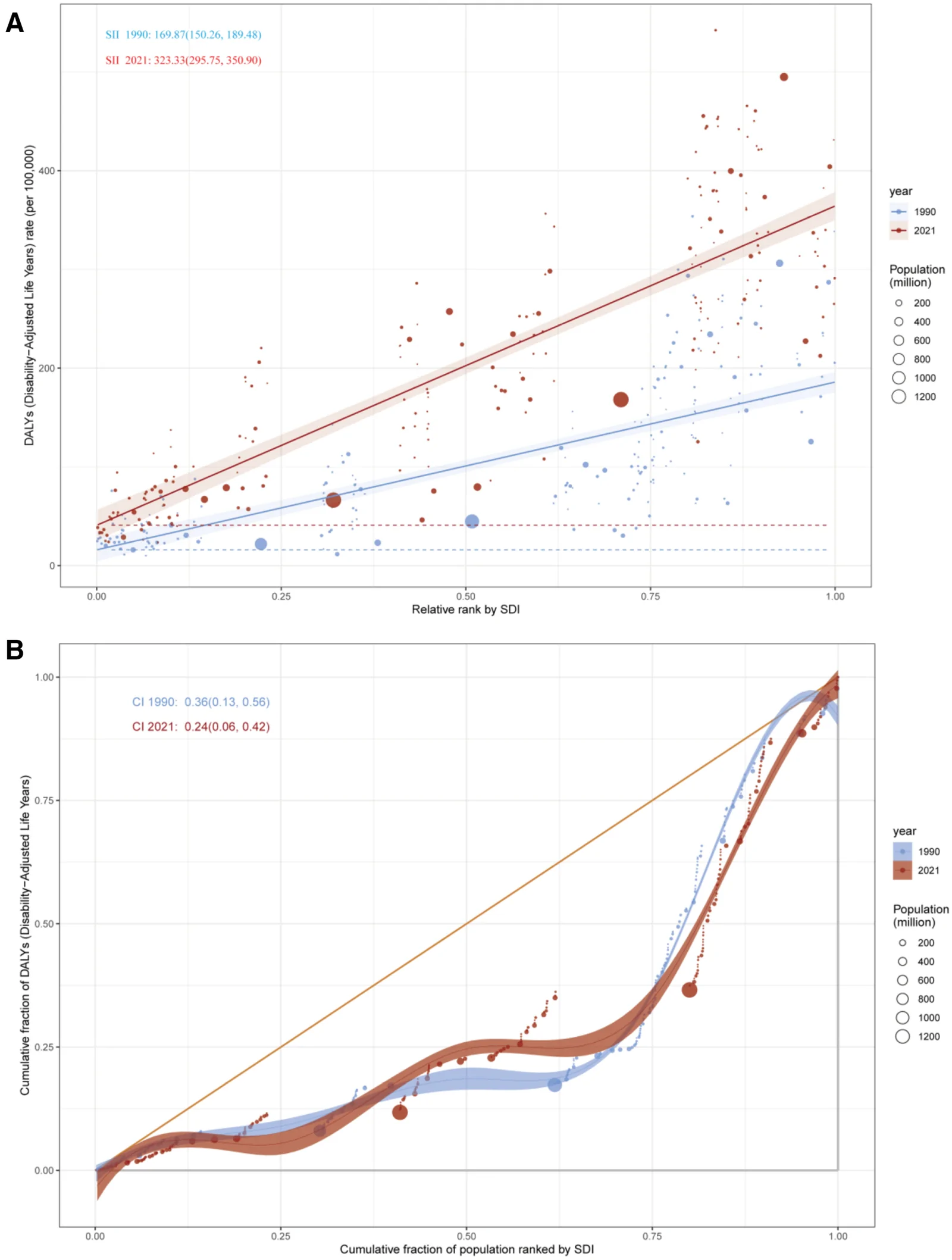
Health inequality regression and concentration curves for MSK diseases attributed to high BMI according to DALYs rate. (A) Health inequality regression for DALYs rate. (B) Concentration curves for DALYs rates. BMI = body mass index, DALYs = disability-adjusted life years, MSK = musculoskeletal.

As shown in Figure 5, we observed significant absolute and relative SDI-related inequalities in DALYs of MSK disorders attributable to high BMI across 204 countries and territories, with countries with higher SDI bearing a disproportionate burden. From 1990 to 2021, absolute inequalities in DALYs of MSK disorders attributable to high BMI were further exacerbated in countries with higher SDI, with the SII increasing from 169.87 (95% CI = 150.26–189.48) to 323.33 (95% CI = 295.75–350.90; Fig. 5A). However, the concentration index of DALYs of MSK disorders attributable to high BMI decreased from 0.36 (95% CI = 0.13–0.56) to 0.24 (95% CI = 0.06–0.42), suggesting that the burden of MSK disorders attributable to high BMI improved somewhat in terms of relative inequality between countries and regions with higher SDI (Fig. 5B).

### 3.5. Prediction of MSK disease burden attributable to high BMI

**Figure 6.**
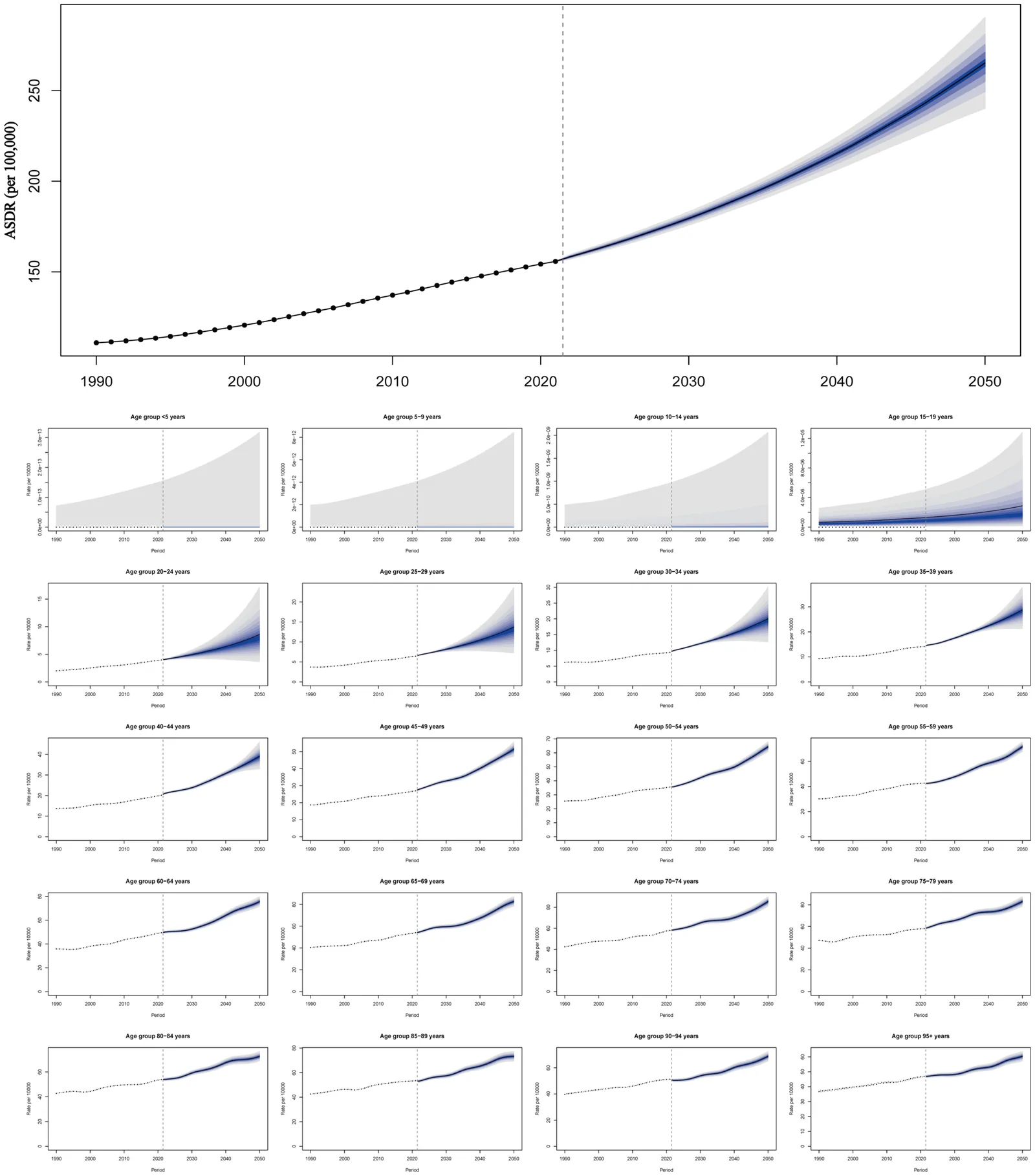
Prediction results of ASDR for MSK disorders attributable to high BMI. ASDR = age-standardized DALYs rate, BMI = body mass index, MSK = musculoskeletal.

Figure 6 shows our future projections of ASDR of MSK disorders attributable to high BMI based on the GBD 2021 study. The BAPC model projections show an increasing trend in ASDR of MSK disorders attributable to high BMI globally from 2022 to 2050, with the global ASDR for MSK disorders attributable to high BMI increasing from 155.33 per 100,000 in 2021 to 265.24 per 100,000 in 2050. Meanwhile, the global ASDR of MSK disorders attributable to high BMI is increasing in all age groups from 2022 to 2050.

## 4. Discussion

MSK disorders are a major challenge to global public health and severely affect the physical and mental health of those affected. The global epidemiologic burden of MSK disease has increased rapidly over the past 30 years.^[7,16]^ Risk factors such as smoking, occupational factors, sedentary lifestyle, genetic factors, excessive alcohol consumption, and high BMI have been widely reported in the previous literature to increase the risk of MSK disease onset and progression.^[17–19]^ Among the many risk factors, high BMI is emerging as an important risk factor for MSK disease, with more far-reaching effects than some other behavioral risk factors such as smoking. Globally, overweight and obesity are on the rise worldwide, along with an increasing burden of obesity-related diseases.^[20]^ Therefore, the impact of high BMI on MSK disease burden requires urgent attention and research. This study innovatively used GBD 2021 data to assess temporal trends and health inequalities in MSK disorders attributable to high BMI. We used the corresponding analytical model and various analytical methods to characterize the burden of MSK diseases attributable to high BMI across geographic spaces, periods, and levels of social development.

Our results found that the burden of MSK disorders attributable to high BMI continues to increase globally, with the ASDR of MSK disorders attributable to high BMI rising from 110.57 per 100,000 in 1999 to 155.33 per 100,000 in 2021. The burden of MSK disorders attributable to high BMI varies by country and region. Our findings show a significant positive association between DALYs of MSK diseases attributable to high BMI and SDI levels, suggesting that the burden of MSK diseases attributable to high BMI is heavier in more developed regions and countries. Cross-country inequality analyses found that DALYs of MSK disorders attributable to high BMI had significant absolute and relative SDI-related inequalities across 204 countries and regions, with countries and regions with higher SDIs bearing a disproportionate share of the burden. It has been shown that more developed and upper-middle-income countries have higher rates of obesity and aging populations compared with less developed and lower-income countries and regions.^[21,22]^ This is an important reason why countries and regions with higher SDI have a higher burden of MSK disorders attributable to high BMI. As people’s dietary habits and lifestyles change markedly, the prevalence of obesity is also rising rapidly, adding to the rapidly increasing burden of obesity-related diseases. It has been reported that unhealthy dietary habits and lifestyles are significantly more likely to lead to the development of obesity, thereby exacerbating the development of MSK disorders.^[23]^ Therefore, we should recognize and pay attention to the skeletal-muscular disease burden associated with high BMI and target specific strategies.

The burden of MSK disorders attributable to high BMI varies significantly by sex and age, with the overall burden being considerably higher in women than in men, particularly in the older age groups. Our findings indicate that DALYs rates of MSK disorders attributable to high BMI first showed an increasing trend with age and then a decreasing trend after reaching a peak. Across all age groups, DALYs of MSK disorders attributable to high BMI were much higher in women than in men, and DALYs peaked in the 55 to 59 age group. This difference may be due to a complex combination of biological, genetic, behavioral, and social factors. Some studies have found that alterations in sex hormones are associated with various changes in body composition, fat distribution, and metabolic disorders.^[24]^ As estrogen decreases throughout menopause, energy expenditure and fat oxidation decrease in women, ultimately leading to an increase in total body fat.^[25]^ In addition, there appear to be gender differences in food preferences and eating behaviors between men and women. One study found that women were more likely to prefer sweet and snack-based comfort foods, such as candy or chocolate, while men were more likely to choose meal-based comfort foods.^[24]^ Another study showed that eating disorder behaviors (such as binge-eating disorder and night eating syndrome) are common risk factors for obesity and that these behaviors are more common in women.^[26]^ These may be important reasons why the overall burden of MSK disorders attributable to high BMI is significantly higher in women than in men. In addition to this, the burden of MSK disorders attributable to high BMI is concentrated in the elderly. MSK diseases are chronic, and their prevalence increases with age. Furthermore, lifestyle changes in older adults as they enter retirement may result in a long-term positive energy balance, leading to excessive accumulation of adipose tissue and accelerating the development of age-related diseases.

The BAPC model prediction indicates that the global disease burden of MSK disorders caused by high BMI will continue to increase, necessitating attention to this issue and the implementation of specific measures. At the same time, corresponding projection studies show that the prevalence of obesity is expected to continue to increase, and the burden of obesity-related diseases will further increase. We should recognize and clarify the severity of MSK disorders attributable to high BMI and take effective, targeted measures. In the future, the focus of reducing the burden of MSK diseases attributable to high BMI will be on early prevention and early intervention. Reasonable and effective weight management programs should be formulated according to the actual situation, dietary structure should be improved, and physical activity should be increased moderately to effectively control the incidence of obesity. In addition to early prevention and intervention, scientific and effective management of MSK disorders attributable to high BMI is essential for improving patient prognosis and quality of life. Multidisciplinary management and care, including psychological support, rehabilitation exercise guidance, and long-term follow-up, should be integrated to effectively improve the quality of life of patients affected by MSK disorders.

The present study provides a detailed and comprehensive analysis and overview of the burden of MSK disorders attributable to high BMI, and despite valuable findings, this study has several limitations that should be recognized. First, the quality and availability of data vary widely across countries and regions, which may result in underestimating or inaccurately reflecting the true burden of disease in these regions. Second, the study was observational and therefore could not determine a causal relationship between high BMI and the development of MSK disorders. Third, our projections of the future burden of MSK disorders attributable to high BMI are based on current trends and patterns and do not take into account potential future changes in other relevant risk factors, shifts in health care policy, and so on. Finally, our study relied on the methodological framework of the GBD study. However, there may be potential biases in the data collection and modeling methods of GBD. Despite the limitations of this study, our study provides valuable insights into studies related to the burden of skeletal-muscular disease attributable to high BMI.

## 5. Conclusion

In summary, our findings suggest that the global burden of MSK disorders attributable to high BMI has been increasing from 1990 to 2021 and is expected to continue to increase in the future. The burden of MSK disorders attributable to high BMI varies markedly across socioeconomic development countries, gender, and age groups. In the future, multifaceted strategies and targeted measures will be needed to effectively manage the burden of MSK disorders attributable to high BMI.

## Acknowledgments

We are grateful to the countless individuals who have contributed to the Global Burden of Disease Study 2021 in various capacities.

## Author contributions

**Conceptualization:** Jiantong Wei, Qingqing Qin, Yaohua Wang.

**Data curation:** Jiantong Wei, Qingqing Qin, Hao Chen, Wenqiang Liang, Yaohua Wang.

**Formal analysis:** Qingqing Qin, Hao Chen, Wenqiang Liang.

**Methodology:** Hao Chen, Wenqiang Liang.

**Writing – original draft:** Jiantong Wei.

**Writing – review & editing:** Yaohua Wang.
